# Serum nutrient profile and dietary patterns as predictors of tumor grade and molecular subtype in breast cancer patients

**DOI:** 10.3389/fnut.2026.1745421

**Published:** 2026-01-26

**Authors:** Xiaohu Sun, Zhihao Yu, Ran Meng, Xin Wang, Xuchen Cao

**Affiliations:** 1 Tianjin Medical University Cancer Institute and Hospital, National Clinical Research Center for Cancer, Tianjin, China; 2 Key Laboratory of Cancer Prevention and Therapy, Tianjin, China; 3 Tianjin’s Clinical Research Center for Cancer, Tianjin, China; 4 Key Laboratory of Breast Cancer Prevention and Therapy, Tianjin Medical University, Ministry of Education, Tianjin, China

**Keywords:** breast cancer, dietary patterns, molecular subtype, oxidative stress, serum nutrients, tumor grade

## Abstract

**Background:**

Breast cancer heterogeneity is influenced by tumor grade, molecular subtype, and modifiable lifestyle factors such as diet and nutritional status. Tumor aggressiveness and oxidative stress may be influenced by dietary habits and serum nutritional profiles, according to new research.

**Aim:**

The purpose of this study was to assess the relationship between oxidative stress markers, dietary patterns, serum nutritional profiles, and breast cancer tumor features, such as tumor grade and molecular subtype.

**Methodology:**

Using validated questionnaires, the tumor grade, molecular subtype, serum nutrients (vitamins, trace elements, lipids, oxidative stress indicators), and food intake of 293 female patients with breast cancer were evaluated in this retrospective analysis. Dietary patterns were found using principal component analysis, and statistical analyses included correlation matrices and logistic regression.

**Results:**

The molecular subtypes of the tumors were Luminal A (38.2%), Luminal B (24.9%), HER2-enriched (21.3%), and triple-negative (15.7%). The tumor grades were Grade I (29.8%), II (45.5%), and III (24.7%). With tumor grade, oxidative stress (MDA) rose and antioxidant nutrients decreased (*p* < 0.01). Plant-based, Western, Mixed, and Mediterranean-like eating patterns were found. While the Mediterranean-like diet was beneficial (OR = 0.60, *p* = 0.041), excessive adherence to the Western diet was linked to increased risks of aggressive tumors (OR = 2.30, *p* = 0.003). Antioxidant nutrients and adherence to the Mediterranean-like diet were shown to be favorably correlated; MDA was positively correlated with the Western pattern.

**Conclusion:**

Antioxidant-rich Mediterranean-like dietary pattern showed inverse association with aggressive tumor features, suggesting potential protective biological relationship while Western dietary pattern was positively associated with oxidative stress and lower circulating antioxidant nutrients. Personalized nutrition methods to improve breast cancer prognosis may be informed by the integration of dietary and biochemical assessment.

## 1 Introduction

Breast cancer continues to be the most common disease diagnosed in the world and a major contributor to cancer-related death in women. The heterogeneity of breast cancer presents difficulties for prognosis and treatment response despite notable advancements in screening and therapy methods. Disease aggressiveness and treatment outcomes are significantly influenced by tumor grade and molecular subtype, which are defined by proliferation markers (such Ki-67) and hormonal receptor status (ER, PR, and HER2). According to new research, breast carcinogenesis, tumor progression, and molecular differentiation are significantly influenced by nutritional status, both in terms of blood nutrient profile and dietary patterns (1, 2). Therefore, finding dietary habits and nutritional indicators that predict tumor characteristics may help us better understand the modifiable factors associated with the prognosis of breast cancer.

Vitamins, trace elements, lipids, and antioxidants are examples of serum nutrients that represent both dietary consumption and metabolic changes in the tumor microenvironment. Micronutrient deficiencies or imbalances, including those in vitamin D, selenium, zinc, and copper, have been shown to affect oxidative stress, DNA repair capability, and immunological regulation, which in turn affects tumor grade and development (1). Furthermore, aggressive molecular subtypes of breast cancer have been linked to disruption of lipid metabolism. Higher tumor grade and the luminal B and HER2-enriched subtypes, which are associated with worse outcomes, were found to be substantially correlated with raised blood triglycerides and LDL cholesterol (2, 3). These results demonstrate the potential use of lipid and nutrition profiles in serum as biochemical predictors of tumor behavior.

It is becoming more widely acknowledged that dietary patterns which comprise the entirety of regularly consumed foods are more significant predictors of illness risk than individual nutrients. Research indicates that “Western” eating habits, which are marked by high consumption of processed foods, red meat, saturated fats, and refined carbs, are linked to elevated levels of oxidative stress and systemic inflammation, both of which encourage the development of tumors (4, 5). On the other hand, following a sensible or Mediterranean-style diet that is high in fruits, vegetables, whole grains, legumes, and seafood has been linked to lower rates of breast cancer, fewer recurrences, and better survival (6, 7). Certain molecular subtypes have also been linked to the inflammatory potential of diet, as measured by indices like the Dietary Inflammatory Index (DII). According to Hayati et al. (5), triple-negative and HER2-positive breast cancers subtypes with greater proliferative potential and a poorer prognosis were more closely linked to pro-inflammatory diets.

Tumor biology is influenced by nutritional and dietary variables through a number of interrelated pathways. Antioxidant micronutrients like vitamin E, vitamin C, and selenium control the expression of genes involved in cell proliferation and apoptosis as well as the levels of reactive oxygen species (ROS) (1). As signaling agents, lipid metabolites influence the generation of inflammatory cytokines and estrogen biosynthesis, which in turn affects receptor expression and subtype differentiation (2, 8). Additionally, dietary habits influence the systemic inflammatory milieu and gut microbiota, which in turn control immune surveillance and estrogen metabolism two crucial processes in the development of breast cancer (7, 9). Further mechanistic evidence is provided by serum metabolomics profiling, as shown by Fang et al. (10), which shows that metabolic changes in amino acid, lipid, and energy pathways can reflect tumor aggressiveness and predict response to medication.

There are still few integrative analyses that look at how blood nutrient profiles and dietary patterns jointly predict tumor grade and molecular subtype in patients who have already been diagnosed, despite the fact that prior research has examined the impact of diet or specific nutrients in breast cancer risk and survival. The majority of the information that is currently accessible, including the systematic review by Bu et al. (4), focusses on pre-diagnostic eating practices and how they affect cancer risk or overall prognosis. Few studies, however, have looked at the simultaneous nutritional and metabolic factors that could account for intra-tumoral heterogeneity at the molecular level. Furthermore, there is little data from East Asian communities, whose dietary practices, nutrient inadequacies, and breast cancer biology are very different from those of Western cohorts. Thus, a thorough assessment that connects blood nutrient indicators to dietary pattern adherence and tumor pathology may close a significant knowledge gap in cancer and precision nutrition.

The goal of the current study is to thoroughly examine the connection between dietary habits, tumor features, and blood nutritional profiles in patients with breast cancer. It specifically aims to assess differences in serum nutritional levels across various tumor grades and molecular subtypes, including vitamins, lipids, and antioxidant enzyme activities. Additionally, the study aims to identify the most common food patterns among individuals and investigate the relationship between tumor aggressiveness and molecular profile and adherence to these patterns. In order to gain a better understanding of their combined impact on the biology of breast cancer, it also seeks to investigate the connections between serum nutritional biomarkers, oxidative stress indicators, and inflammatory markers. The ultimate goal of this research is to determine whether particular dietary habits and serum nutrient imbalances can function as trustworthy indicators of tumor grade and molecular subtype. This will lay the groundwork for creating individualized nutrition-based strategies to enhance prognosis and clinical outcomes in patients with breast cancer.

## 2 Methodology

### 2.1 Study design and setting

The Department of Oncology carried out this retrospective analytical analysis. The purpose of the study was to investigate the relationship between tumor grade and molecular subtype in breast cancer patients and blood nutritional profiles and dietary habits. From January 2020 to December 2024, information was gathered from previously operational institutional electronic medical records and laboratory information systems. Dietary Intake data were gathered three to 6 months after the histological confirmation of the diagnosis of breast cancer and before the start of endocrine therapy, chemotherapy, or radiation. Prior to any systemic treatment, baseline blood samples were collected for biochemical and oxidative stress indicators. This research was approved by the Ethics Committee of Tianjin Cancer Hospital, No. Ek2019086. To guarantee confidentiality and adherence to the specified ethical standards, all patient data was anonymized before analysis.

### 2.2 Study population

#### 2.2.1 Inclusion criteria

Female patients aged 30–75 years, with histologically confirmed primary breast carcinoma, were included. Complete serum biochemical and nutritional profile data, as well as verified dietary records, were to be available within 6 months of the diagnosis in order to be eligible. To enable molecular classification, patients with established human epidermal growth factor receptor 2 (HER2), progesterone receptor (PR), and estrogen receptor (ER) status were included.

#### 2.2.2 Exclusion criteria

Patients with missing medical or nutritional information, those with metastatic disease or secondary malignancies, or those who had received chemotherapy, radiation, or hormone therapy before serum sample were excluded. Additionally, people with long-term inflammatory or metabolic conditions (such as diabetes mellitus, renal failure, or autoimmune diseases) that can interfere with nutritional or biochemical assessments were not included.

### 2.3 Data collection

Dietary assessment, blood sampling, and clinic pathological data collection were performed at baseline during the diagnostic work-up period. Data were extracted from laboratory databases and hospital records using a standardized extraction form. The dataset included tumor characteristics (size, grade, stage, and receptor status), demographic variables (age, BMI, and menopausal status), and serum biochemical parameters (vitamins, minerals, lipid profile, glucose, and oxidative stress markers). Dietary intake was assessed using food frequency questionnaires (FFQs) and validated 3-day dietary recalls, with data entered into patient nutrition records. Data collected between 2020 and 2024 provided a comprehensive characterization of patient profiles and treatment-naïve biochemical status.

### 2.4 Serum biochemical and nutrient profile assessment

Standardized laboratory techniques were used to analyze serum samples obtained during routine pre-treatment evaluation. Included were the following parameters:

- Fasting venous blood samples were collected in the morning after a minimum 10-h fast prior to invasive diagnostic procedures or treatment initiation.
- Vitamins A, D, E, B12, C, and folate (measured using chemiluminescent immunoassays)
- Minerals: iron, zinc, selenium, calcium, and magnesium (as determined by atomic absorption spectrophotometry)
- Glycaemic parameters: insulin and fasting glucose (quantified using ELISA)
- Antioxidant markers: Superoxide dismutase (SOD), and malondialdehyde (MDA) levels (spectrophotometric tests)

The Clinical and Laboratory Standards Institute (CLSI) and the World Health Organization’s standards served as the foundation for reference ranges. The use of certified reference materials and duplicate sample analyses (coefficient of variation <5%) were among the internal and external quality control protocols that were upheld.

#### 2.4.1 Dietary assessment

Dietary consumption was evaluated through semi-quantitative Food Frequency Questionnaire (FFQ) adapted from the Chinese Health and Nutrition Survey (CHNS), previously validated in Chinese adult populations, which was based on earlier validated questionnaires that were used in East Asian communities. The FFQ was a measure of the usual intake of dietary items during the past 12 months, and covered the major food categories, including fruits, vegetables, whole grains, red and processed meats, sweets and fast foods, dairy products, and fats and oils. Estimation of portions was done by using standard household measures and food models. Besides that, the trained dietitians carried out 3-day dietary recalls (two working days and one rest day) to approximate the nutrient intake, as well as to assist in the validation of FFQ-based data.

#### 2.4.2 Dietary pattern analysis

Principal component analysis (PCA) was used to derive dietary patterns based on energy-adjusted intakes of food groups based on grams per day. The residual method was used to do the energy adjustment. The Kaiser–Meyer–Olkin (KMO) index of sampling adequacy (KMO = 0.83) and significant Bartletts test of sphericity (*p* = 0.001) confirmed the suitability of the data to PCA. The factors were kept considering the eigenvalues above 1.0, scree plot reviews, and interpretability. The Varimax rotation was used to maximize the interpretability of the factors and the factor loading ≥0.40 was deemed significant. Eating patterns were named in terms of the prevalent food group loadings and are known as plant-based, Western, mixed, and Mediterranean-like dietary patterns.

Four main eating trends were found and classified as follows:

- *Plant-based pattern*: Consuming a lot of fruits, vegetables, whole grains, and legumes
- *High-fat/western pattern*: Rich in processed meals, saturated fats, and red meat
- Moderate consumption of both plant and animal goods is a mixed pattern.
- *Mediterranean-like dietary pattern*: Packed with seafood, vegetables, nuts, and olive oil

For further study, participants were divided into tertiles (low, medium, high adherence) based on how closely they adhered to each pattern.

### 2.5 Tumor grade and molecular subtype classification

Tumors were categorized as Grade I (well-differentiated), Grade II (moderately differentiated), or Grade III (poorly differentiated) using the Nottingham variation of the Scarff-Bloom-Richardson system for histopathological grading. Based on the immunohistochemistry (IHC) expression of ER, PR, HER2, and Ki-67 markers, molecular subtypes were categorized into:

- Luminal A (low Ki-67, HER2–, ER+/PR+)
- Luminal B (high Ki-67, HER2+/−, ER+/PR+)
- HER2-enriched (HER2+, ER–/PR–)
- ER–/PR–/HER2– triple-negative

Fluorescence *in situ* hybridisation (FISH) was used to confirm HER2 equivocal instances on IHC.

### 2.6 Statistical analysis

SPSS version 28.0 were used to analyze the data. Continuous variables were summarized using means with standard deviations or medians with interquartile ranges, while categorical variables were summarized using frequencies and percentages. The Pearson or Spearman correlation coefficients, as the case is, were used to measure correlations between dietary pattern scores and biochemical markers. To assess the relationship between dietary patterns and tumor grade and molecular subtypes, logistic regression models have been used. Ordinal logistic regression was used to test tumor grade, whereas multinomial logistic regression models were used to test molecular subtypes. Multivariate regressions were checked with age, body mass index (BMI), menopause status, total energy consumption, physical activities, smoking status, alcohol consumption, level of education, tumor size, and tumor stage. Odds ratios (ORs) along with 95 percent confidence intervals (CIs) were provided and statistical significance value was established at *p* < 0.05.

## 3 Results

### 3.1 Participants characteristics

A total of 293 breast cancer patients were included. Mean age: 52.4 ± 9.8 years; mean BMI: 27.6 ± 4.3 kg/m^**2**^. Most were postmenopausal (58%). Tumor grades were distributed as follows: Grade I (29.75%), Grade II (45.53%), Grade III (24.72%). Molecular subtypes: Luminal A (38.2%), Luminal B (24.85%), HER2-enriched (21.3%), Triple-negative (15.65%) (Table 1).

**Table 1:** Baseline characteristics of patients.

Variable	Mean ± SD/%
Age (years)	52.4 ± 9.8
BMI (kg/m^2^)	27.6 ± 4.3
Postmenopausal	58%
Grade I	29.75%
Grade II	45.53%
Grade III	24.72%
Luminal A	38.2%
Luminal B	24.85%
HER2-enriched	21.3%
Triple-negative	15.65%

The values are given in the form of mean ± standard deviation, or percentage (%). BMI was determined as weight (kg)/height^2^(m^2^). The grade of tumor was determined using the Nottingham system of grading. Immunohistochemically evaluation of ER, PR, HER2 and Ki-67 was used to define molecular subtypes. The values are all crude (unadjusted). Table 1 was not tested in regard to any hypothesis.

### 3.2 Serum nutrient profiles across tumor grades

**Figure 1:**
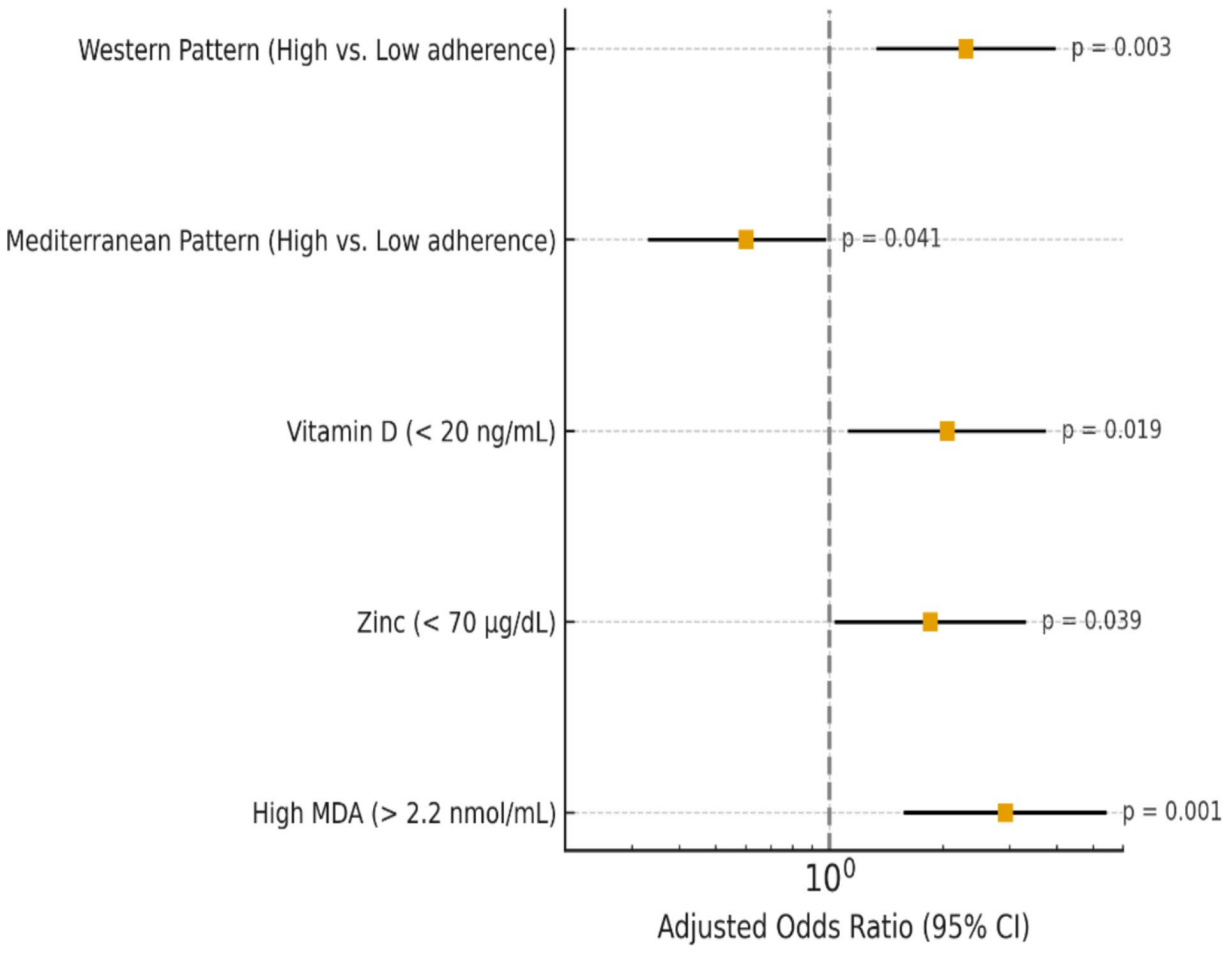
Multivariate logistic regression predictors of high tumor grade in breast cancer patients.

The comparison of serum nutritional indicators in patients with breast cancer across various tumor grades is shown in Table 2. The oxidative stress marker MDA increases considerably from Grade I to Grade III (*p* < 0.001), yet there is a definite trend indicating a progressive decline in antioxidant micronutrients (Vitamin D, E, A, C, zinc, selenium, and magnesium) with increasing tumor grade. Similarly, as a tumor progresses, SOD activity, a measure of antioxidant defense, significantly declines (*p* = 0.003). These results point to an imbalance between pro-oxidant and antioxidant systems in the pathogenesis of breast cancer, as increasing tumor grade is linked to nutrient depletion and enhanced oxidative stress (Figure 1).

**Table 2:** Comparison of serum nutrient markers according to tumor grade.

Biomarker	Grade I	Grade II	Grade III	*p-*value
Vitamin D (ng/mL)	29 ± 6	23 ± 6	18 ± 4.8	0.001**
Zinc (μg/dL)	91 ± 14	80 ± 12.1	69 ± 10.2	0.009**
Selenium (μg/L)	121 ± 23	111 ± 20.2	99 ± 18	0.028**
Magnesium (mg/dL)	2.1 ± 0.2	2 ± 0.2	1.7 ± 0.2	0.004**
Vitamin E (μg/mL)	11 ± 2	9 ± 2.0	7.5 ± 1.8	0.002**
Vitamin A (μg/dL)	59 ± 11	54 ± 10.2	47 ± 9.3	0.015**
Vitamin C (mg/dL)	1.4 ± 0.3	1.2 ± 0.3	0.9 ± 0.3	0.001**
MDA (nmol/mL)	2.0 ± 0.5	2.4 ± 0.5	3.0 ± 0.6	< 0.001**
SOD (U/mL)	2.6 ± 0.6	2.2 ± 0.6	1.8 ± 0.6	0.003**

*p < 0.05 significant; *p < 0.01 highly significant.

Values are mean ± SD. Sample sizes are approximate due to rounding. Vitamin D deficiency was defined as <20 ng/mL, zinc deficiency as <70 μg/dL, elevated MDA as >2.2 nmol/mL, and low SOD activity as <2.0 U/mL. Group comparisons were performed using one-way ANOVA.

MDA, malondialdehyde; SOD, superoxide dismutase.

### 3.3 Dietary patterns and variance explained

Table 3 shows four distinct dietary patterns identified through principal component analysis: Plant-Based, Western-type, Mixed, and Mediterranean-like. The *Plant-Based* pattern is rich in fruits, vegetables, whole grains, and legumes. The *Western* pattern features high intakes of red/processed meat, sweets, and refined grains. The *Mixed* pattern includes moderate consumption of dairy, legumes, and refined grains, reflecting a blend of healthy and less healthy foods. The *Mediterranean-like* pattern is characterized by high loadings for olive oil, fish, nuts, fruits, and whole grains. These patterns represent diverse dietary behaviors potentially linked to variations in nutrient status and breast cancer characteristics. PCA extracted four major dietary patterns explaining 75% of the total variance.

**Table 3:** Factor loadings for major dietary patterns.

Food group	Plant-based	Western	Mixed	Mediterranean
Fruits & vegetables	**0.81**	−0.09	0.39	0.74
Red/processed meat	−0.22	**0.78**	0.35	−0.18
Whole grains	0.77	0.21	0.42	0.69
Sweets/fast food	−0.15	**0.83**	0.29	−0.16
Olive oil/fish/nuts	**0.52**	−0.23	0.33	**0.87**
Dairy products	0.34	0.47	**0.61**	0.29
Legumes & pulses	**0.74**	0.12	0.58	0.65
Refined grains	−0.09	**0.71**	0.46	−0.11

Bold values represent statically meaningful factor loadings.

Dietary intake was energy-adjusted using the residual method and expressed as grams/day. PCA was conducted on standardized variables. Kaiser–Meyer–Olkin statistic = 0.83 (0.80–0.89); Bartlett’s test of sphericity *p* < 0.001. Factor loadings ≥0.40 are shown in bold.

### 3.4 Association between dietary patterns and tumor characteristics

High adherence to the Western type diet significantly raised the odds of aggressive molecular subtypes and higher tumor grade (OR = 2.30, *p* = 0.003), whereas the Mediterranean-like diet had a protective effect (OR = 0.60, *p* = 0.041), according to Table 4. Elevated MDA significantly enhanced the chance of advanced tumor grade (*p* = 0.001), and low vitamin D and zinc levels were linked to increased tumor aggressiveness. In general, more aggressive breast cancer characteristics were associated with pro-inflammatory diets and lower antioxidant status. The observed associations remained statistically significant after additional adjustment for physical activity and smoking status. An ordinal logistic regression yielded odds ratios (OR) and 95% confidence intervals (CI) of tumor grade, and a multinomial logistic regression of molecular subtype. *n* = 268 after exclusion of missing covariates was determined as the final analytical sample. Every estimation is corrected to age, body mass, menopause, total energy consumption, physical activity, status of smoking, consumption of alcohol, level of education, tumor size, and tumor stage.

**Table 4:** Multivariable regression analyses for dietary patterns and serum biomarkers in relation to breast cancer outcomes.

Variable	Category/cut-off (units)	Adjusted OR (95% CI)	Model type	Statistical test	*p*-value	*n* in Category
Western dietary pattern score	High vs. low adherence	2.30 (1.33–3.98)	Multinomial	Wald *χ*^2^	**0.003**	High *n* = 146 / Low *n* = 147
Mediterranean dietary pattern score	High vs. low adherence	0.60 (0.33–0.98)	Multinomial	Wald *χ*^2^	*0.041*	High *n* = 139 / Low n = 154
Vitamin D	Deficiency <20 ng/mL	2.05 (1.12–3.75)	Ordinal	Wald *χ*^2^	*0.019*	Deficient *n* = 128
Zinc	<75 μg/dL	1.85 (1.03–3.32)	Ordinal	Wald χ^2^	*0.039*	Low *n* = 102
Selenium	<100 μg/L	1.72 (0.95–3.10)	Ordinal	Wald *χ*^2^	0.067	Low *n* = 96
MDA	>2.2 nmol/mL	2.92 (1.57–5.43)	Ordinal	Wald *χ*^2^	**0.001**	High *n* = 137
SOD activity	<2.0 U/mL	2.11 (1.18–3.79)	Ordinal	Wald *χ*^2^	*0.012*	Low *n* = 110

Bold values represent significant correlation coefficient.

### 3.5 Correlation matrix

The correlation analysis indicated that there were a number of statistically significant relationships between consuming dietary patterns, nutritional biomarkers and oxidative stress indicators (Table 5). Biomarkers associated with antioxidants, such as vitamin D, zinc, selenium, and superoxide dismutase (SOD) activity, exhibited a high level of significant positive intercorrelations and this indicates their interdependence with each other with regard to ensuring antioxidant defence against oxidative stress. The positive relation between the serum vitamin D levels and the SOD activity indicated that there might be the relationship between the vitamin D status and the endogenous antioxidant capacity. Conversely, malondialdehyde (MDA) levels were found to be inversely related with antioxidant nutrients and SOD (*r* = −0.48, *p* < 0.01) therefore, showing higher oxidative stress with lower antioxidant protection. An increase in MDA was also positively correlated with the tumor grade and is an indication of increased oxidative load in more aggressive tumors. An analysis of dietary patterns revealed that an increase in adherence to Western dietary pattern was significantly correlated with increased levels of MDA (*r* = 0.39, *p* < 0.01) and negatively correlated with antioxidant markers, and dietary pattern adherence to a Mediterranean-like pattern was found to positively correlate with vitamin D, zinc, selenium and SOD. These results indicate that the Western type dietary practices have the potential of increasing oxidative stress and nutrient wastage, whereas the antioxidant-dense dietary practices favor a more desirable nutritional and oxidative status.

**Table 5:** Correlation matrix of serum nutrients, oxidative stress markers, and dietary patterns in breast cancer patients.

Variables	Vitamin D	Zinc	Selenium	MDA	SOD	Western pattern	Mediterranean pattern
Vitamin D	1	0.42**	0.38**	−0.33**	0.41**	−0.29*	0.35**
Zinc	0.42**	1	0.47**	−0.31**	0.39**	−0.25*	0.28*
Selenium	0.38**	0.47**	1	−0.36**	0.44**	−0.22	0.33**
MDA	−0.33**	−0.31**	−0.36**	1	−0.48**	0.39**	−0.32**
SOD	0.41**	0.39**	0.44**	−0.48**	1	−0.35**	0.36**
Western type pattern	−0.29*	−0.25*	−0.22	0.39**	−0.35**	1	−0.41**
Mediterranean-like pattern	0.35**	0.28*	0.33**	−0.32**	0.36**	−0.41**	1

*Shows significant; **Highly significant.

**Figure 2:**
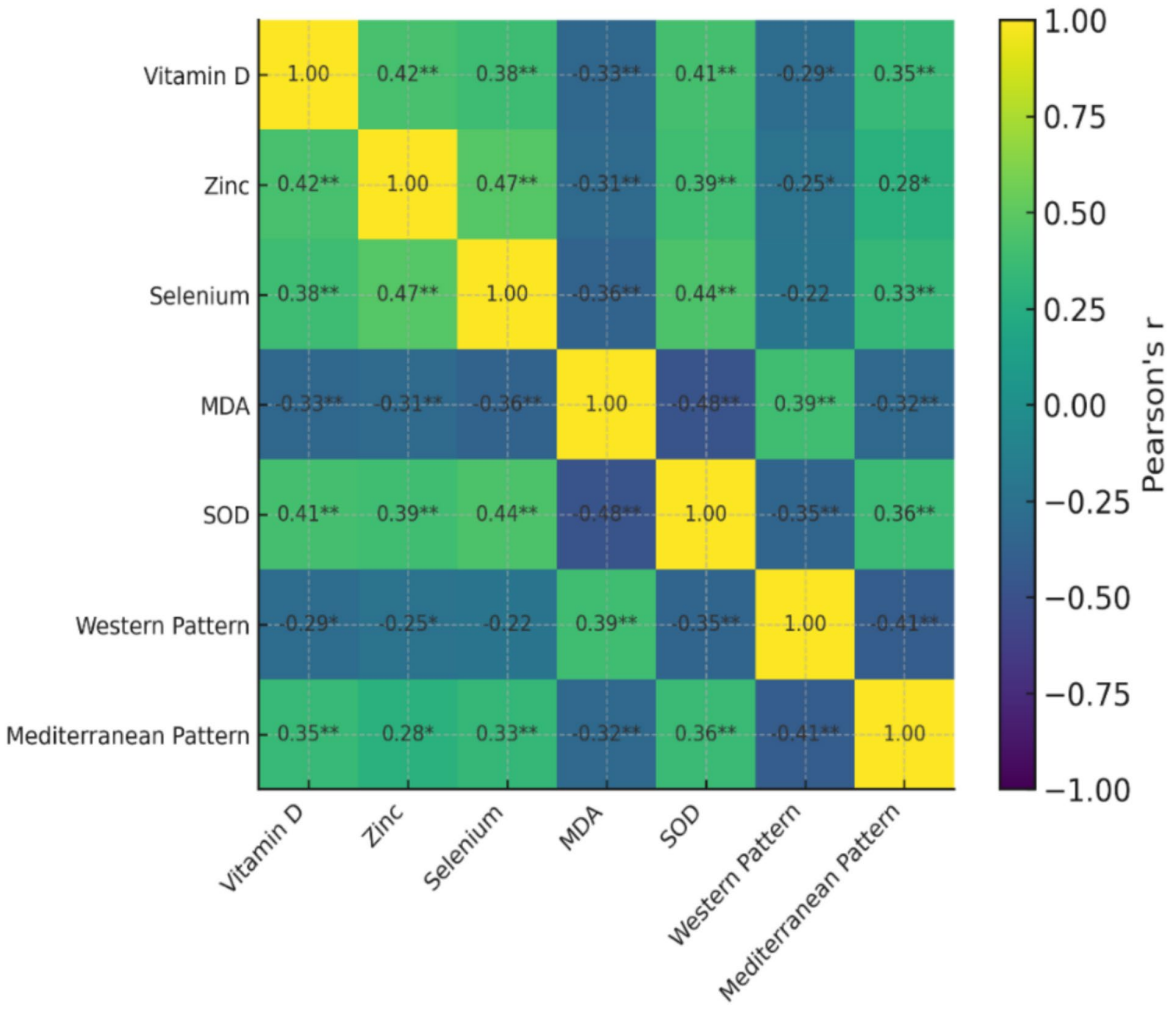
Heatmap showing correlations between serum nutrients, oxidative stress markers, and dietary patterns in breast cancer patients.

An association heatmap between food pattern scores, blood nutritional indicators, oxidative stress markers, and tumor features is shown in Figure 2. When necessary, Pearson or Spearman methods were used to calculate correlation coefficients. The degree and direction of connections are shown in color intensity; stronger links are indicated by darker tones. Correlations that are statistically significant (*p* < 0.05) are shown.

## 4 Discussion

A total of 293 patients with breast cancer were examined in this study; their mean age was 52.4 ± 9.8 years, and their mean BMI was 27.6 ± 4.3 kg/m^2^. Tumor grades and molecular subtypes were distributed as follows: Grade I (29.75%), Grade II (45.53%), Grade III (24.72%), Luminal A (38.2%), Luminal B (24.85%), HER2-enriched (21.3%), and Triple-negative (15.65%). The majority were postmenopausal (58%). Like other observational studies in early- and mid-stage breast cancer populations, these baseline characteristics constitute a representative cohort of individuals with breast cancer. The Luminal A subtype and Grade II tumor predominance are consistent with research by Faur et al. (2), which showed that luminal subtypes make up most cases of breast cancer and are typically linked to less aggressive behavior than HER2-enriched and triple-negative subtypes. Additionally, our distribution is similar to that of León-Carreño et al. (11), who highlighted the global consistency of subtype prevalence by noting Luminal A as the most common subtype in their Colombian cohort despite observing variation in molecular subtypes between populations.

It is interesting to note that our cohort’s anthropometric and demographic characteristics match those of Trestini et al. (12), who found that early-stage breast cancer patients had similar mean ages and BMIs. Tailored nutritional interventions can improve adherence to dietary guidelines, lower BMI, and positively modulate serum metabolic biomarkers in these populations, according to Trestini and colleagues. This suggests that baseline nutritional status and lifestyle factors are critical for the prognosis of breast cancer and overall metabolic health. Therefore, when assessing the influence of food and serum nutritional status on breast cancer outcomes, our results highlight the importance of taking into account patient variables, such as age, menopausal status, BMI, tumor grade, and molecular subtype. Our cohort’s resemblance to earlier research validates the generalizability of our findings and lays the groundwork for incorporating nutritional therapies customized to certain tumor grades and molecular subtypes, as advised by evidence-based nutritional oncology practices.

The current investigation showed a strong correlation between oxidative stress markers, tumor grade, and serum nutritional profiles in individuals with breast cancer. As tumor grade grew, antioxidant micronutrients such as vitamins D, E, A, C, zinc, selenium, and magnesium gradually decreased while oxidative stress, as shown by MDA levels, dramatically increased. Consequently, SOD activity, a crucial enzymatic antioxidant defense, declined. These results show that when tumors become more aggressive, the balance between pro-oxidant and antioxidant systems is upset, which is in line with earlier research showing that decreased antioxidant capacity accelerates tumor growth (13). In a similar vein, León-Carreño et al. (11) documented metabolic changes in individuals with breast cancer, with more aggressive subtypes showing increased oxidative stress and altered nutritional profiles, confirming the significance of oxidative imbalance in the biology of breast tumors. Four different dietary patterns: Plant-Based, Western, Mixed, and Mediterranean accounted for 75% of the overall variance, according to the analysis. While the Western type pattern was dominated by red/processed meats, sweets, and refined grains, the Plant-Based and Mediterranean-like patterns were rich in fruits, vegetables, whole grains, legumes, and healthy fats. The intermediate consumption of both healthy and unhealthy foods was represented in the mixed pattern. These results are consistent with earlier studies that indicate favorable tumor features and decreased proliferative activity are linked to dietary patterns with increased antioxidant content (13, 14). On the other hand, as noted by Prasetiyo et al. (15) and León-Carreño et al. (11), Western type dietary practices, which are typified by pro-inflammatory and nutrient-poor foods, have been associated with increased tumor aggressiveness and bad metabolic profiles.

In contrast to the Mediterranean-like pattern, which showed a protective effect (OR = 0.60, *p* = 0.041), our study showed that excessive adherence to the Western type dietary pattern significantly raised the odds of higher tumor grade and aggressive molecular subtypes (OR = 2.30, *p* = 0.003). In a similar vein, increased MDA levels and deficits in zinc and vitamin D were linked to more aggressive tumor characteristics, underscoring the combined effect of lower antioxidant status and pro-inflammatory diets on the development of breast cancer. These results are consistent with earlier studies that highlight the impact of nutrition and food on the prognosis of breast cancer. Romanos-Nanclares et al. (16) found a clear correlation between an elevated risk of aggressive breast cancer subtypes and pro-inflammatory dietary patterns that are high in processed foods, red meat, and refined carbohydrates. This finding supports our findings about the negative consequences of the Western diet. The importance of including dietary and biochemical indicators into prognostic models was further supported by Qu et al. (17) and Wei et al. (18), who showed that nutritional and inflammation-based biomarkers could accurately predict pathological response and survival in breast cancer patients.

Additionally, Trestini et al. (12) showed that in patients with early-stage breast cancer, customized nutritional interventions enhanced adherence to healthy dietary standards and optimized blood metabolic indicators, indicating that enhancing food quality may reduce tumor aggressiveness. In line with our findings that low antioxidant nutrients and elevated oxidative stress were associated with greater tumor grade and more aggressive molecular subtypes, Zhang et al. (19) also highlighted that combined nutrition-inflammation indicators serve as robust predictors of clinical outcomes.

Strong correlations between oxidative stress markers, dietary habits, and serum antioxidant nutrients were found in individuals with breast cancer. While MDA, a marker of lipid peroxidation, was negatively correlated with vitamin D, zinc, selenium, and SOD activity (*r* = −0.48, *p* < 0.01), indicating increased oxidative stress, positive correlations among these micronutrients show a coordinated role in maintaining antioxidant defense. These biochemical markers were clearly associated with dietary trends. The Western type pattern showed a negative correlation with antioxidant nutrients and a positive correlation with MDA, indicating that nutrient-poor, pro-inflammatory diets may worsen oxidative stress and nutritional depletion. On the other hand, following the Mediterranean-like pattern was inversely correlated with MDA and positively correlated with antioxidant indicators, suggesting that diets high in antioxidants support the maintenance of favorable metabolic and oxidative profiles. These results are in line with earlier research. Diet-mediated inflammation and nutritional status have a major impact on clinical outcomes in breast cancer survivors, according to Pannu and Constantinou (20). Similarly, healthy eating habits high in fruits, vegetables, and unsaturated fats are linked to lower oxidative stress and improved prognosis across molecular subtypes, according to Hirko et al. (21) and Castro-Espin et al. (9). Asad et al. (22) and Romanos-Nanclares et al. (23), who showed that pro-inflammatory and insulinemia diets contribute to more aggressive breast cancer phenotypes while antioxidant-rich dietary patterns improve metabolic resilience, further support the interaction between dietary behavior, nutrient status, and oxidative stress. Additionally, Castro-Espin and Agudo (24) observed that dietary therapies that increase antioxidant intake can improve breast cancer patients’ survival results.

### 4.1 Reverse causality and temporal considerations

Since the study was retrospective, dietary consumption was determined post-diagnosis of breast cancer, and this creates the potential of reverse causality. Even though the dietary data were gathered before the treatment on the cancer commenced, it cannot be totally ruled out that the dietary behavior would change after the diagnosis. Thus, the identified relationships between dieting habits and dietary nutritional biomarkers, oxidative stress indicators, and tumor features reflect associative statistical relationships influenced by tumor-related metabolic demand; causal direction cannot be determined in this design. Future research in prospective cohort studies and intervention trials should be considered to explicate the time and mechanism addressing the relationships between diet, oxidative stress, and breast cancer progression. Additionally, rather than food insufficiency being the main cause of tumor aggressiveness, it is conceivable that physiologically aggressive tumors may contribute to systemic nutrient depletion through increased metabolic demand and oxidative burden.

### 4.2 Strengths

This study combines biochemical, nutritional, and clinical data to provide a thorough evaluation of the link between serum nutrient profiles, food patterns, and breast cancer tumor features. The statistical power and generalizability of the results are improved by the comparatively large sample size of 293 patients. A comprehensive assessment of diet rather than individual nutrients, reflecting actual eating habits, was made possible by the use of principal component analysis to discover significant dietary patterns. Furthermore, a multifaceted view of how nutritional status may affect tumor grade and molecular subtypes is provided by the concurrent examination of oxidative stress indicators, antioxidant micronutrients, and dietary adherence. The study’s clinical significance is strengthened by the inclusion of both tumor grade and molecular subtypes as outcome indicators.

### 4.3 Limitations

The study contains a number of shortcomings despite its advantages. The cross-sectional methodology makes it more difficult to determine whether food habits, nutritional status, and tumor aggressiveness are causally related. Self-reported methods were used to measure dietary intake, which could lead to misreporting and recall bias. Serum nutrient levels are a single time-point assessment that might not accurately reflect changes or long-term nutritional status. Furthermore, additional confounding variables that could affect tumor features and nutritional status were not completely controlled, including genetic predisposition, physical activity, and comorbidities. Lastly, the results may not be as applicable to larger, ethnically varied groups because the study population was selected from a particular geographic area.

### 4.4 Future perspective

In order to determine the causal links between dietary patterns, serum nutrient profiles, and the advancement of breast cancer, future research should concentrate on prospective and longitudinal studies. The efficacy of nutrition-based approaches, especially diets high in antioxidants and low in inflammation, in lowering tumor aggressiveness or enhancing treatment outcomes may be ascertained through intervention trials. Deeper understanding of the molecular pathways connecting nutrition and tumor biology may be obtained by combining metabolomics and genomic profiling with dietary and biochemical evaluations. Furthermore, creating customized dietary therapies based on particular tumor subtypes and grades may improve breast cancer patients’ survival and clinical results.

## 5 Conclusion

This study shows that while antioxidant-rich diets like the Mediterranean-like pattern have a protective effect, pro-inflammatory Western type dietary patterns, elevated oxidative stress, and serum nutrient depletion are linked to higher tumor grades and more aggressive molecular subtypes in breast cancer patients. These results highlight the significance of assessing blood nutritional status and eating habits as possible tumor biology predictions. A viable strategy to enhance prognosis and promote general patient health may be to integrate nutrition-based evaluations and treatments into the treatment of breast cancer.

## Data Availability

The raw data supporting the conclusions of this article will be made available by the authors, without undue reservation.
